# Supplementary material for: Estrogenic endocrine disruptor exposure directly impacts erectile function
Source: Commun Biol. 2024 Apr 2;7:403. doi: 10.1038/s42003-024-06048-1 (PMC10987563; doi:10.1038/s42003-024-06048-1)
Supplement: Supplementary file 2 — Supplementary information [file 42003_2024_6048_MOESM2_ESM.pdf]

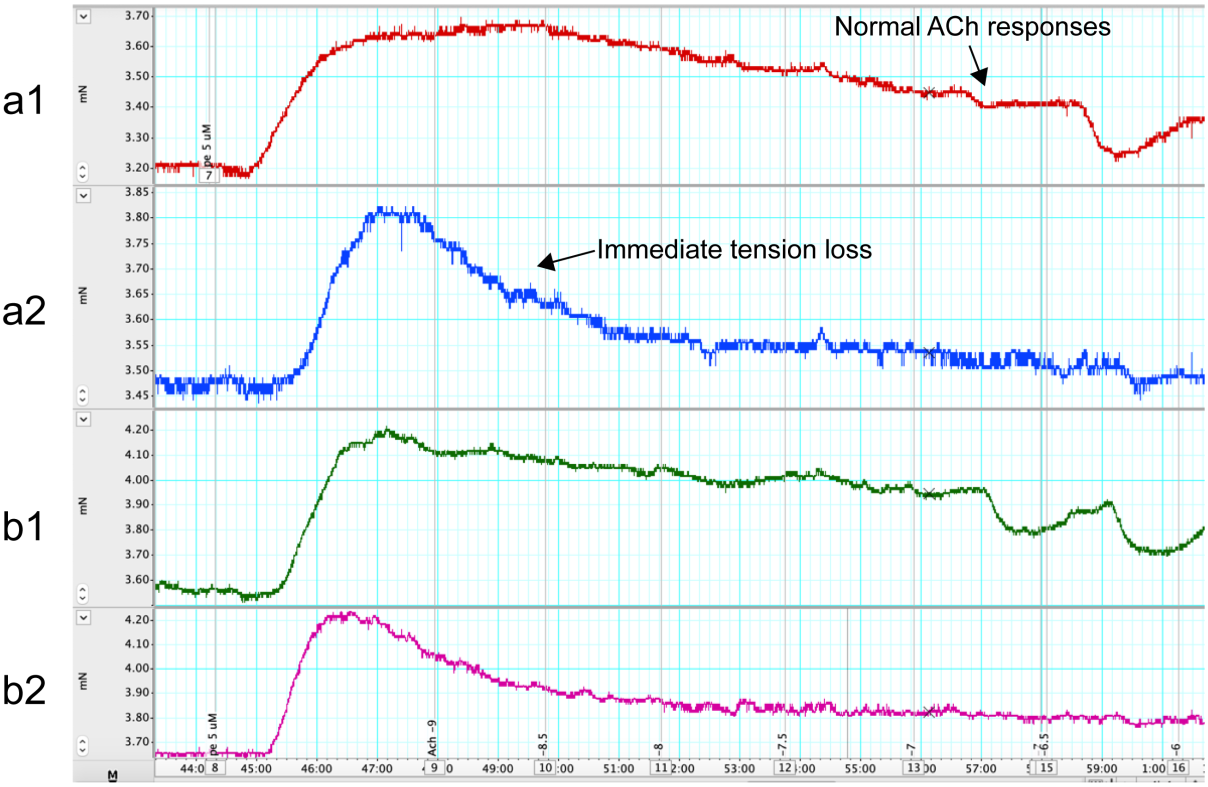

Supplementary Figure 1: ACh-mediated relaxation response of normal CC samples incubated in DES at 10 μM.

Raw wire myograph data showing tension in mN (*y* axis) in real time (*x* axis). **a** and **b:** Each represents two CC samples from one wild-type mouse. Channels **a1** and **b1** (red and green) contain control sample CCs. Reductions in force represent normal responses to acetylcholine (ACh). Channels **a2** and **b2** (blue and pink) contain CCs incubated in DES at 10 μM ($DES_{direct-10}$) which were both dysfunctional as they could not maintain tension, therefore not assessable for ACh-mediated relaxation responses.

Supplementary Table 1: Total wire myography results

Responses of the corpus cavernosum (CC) and mesenteric arteries of various treatment groups to acetylcholine (ACh), sodium nitroprusside (SNP), U46619 and Phenylephrine (PE) in the DES_{water}, $DES_{direct-5}$, $Gen_{direct-20}$ and corresponding control groups. These are calculated as sensitivity ($-logEC_{50}$), max response (% reduction of PE-induced contraction or % of KPSS-induced contraction) and overall response (Area Under the Curve [AUC]). Data expressed as mean ± standard error of the mean (SEM) provided with sample number (n). P values (P) provided for each statistical analysis with the type of statistical test in brackets. Significance at $P < 0.05$.

Tissue	Sample	Drug assay	Sensitivity (-logEC$_{50}$)	P value (statistical test)	Maximal relaxation/contraction (%)	P value (statistical test)	Overall relaxation/contraction (Area Under the Curve [AUC])	P value (statistical test)
CC	DES$_{water}$ control	ACh	7.71 ± 0.07, n = 11	0.13 (Mann Whitney test)	101.3 ± 4.61, n = 11	0.89 (unpaired, two-tailed t-test)	206.6 ± 13.46, n = 11	0.43 (unpaired, two-tailed t-test)
	DES$_{water}$		7.61 ± 0.06, n = 9		102.3 ± 5.56, n = 10		190.7 ± 14.85, n = 10	
	DES$_{water}$ control	SNP	6.41 ± 0.09, n = 11	0.74 (unpaired, two-tailed t-test)	92.77 ± 4.87, n = 11	0.99 (unpaired, two-tailed t-test)	123.2 ± 10.48, n = 11	0.84 (unpaired, two-tailed t-test)
	DES$_{water}$		6.37 ± 0.05, n = 10		92.82 ± 5.33, n = 10		120.5 ± 7.17, n = 10	
	DES$_{water}$ control	U46619	7.22 ± 0.06, n = 8	0.003 (unpaired, two-tailed t-test)	121.7 ± 8.73, n = 8	0.78 (unpaired, two-tailed t-test)	143.8 ± 11.06, n = 8	0.31 (unpaired, two-tailed t-test)
	DES$_{water}$		7.48 ± 0.03, n = 8		117.7 ± 10.61, n = 8		166.8 ± 18.91, n = 8	
	DES$_{direct-5}$ control	ACh	7.31 ± 0.06, n = 9	0.0001 (paired, two-tailed t-test)	91.11 ± 6.36, n = 10	0.26 (paired, two-tailed t-test)	162.9 ± 15.81, n = 10	0.32 (Wilcoxon matched pairs test)
	DES$_{direct-5}$		6.61 ± 0.08, n = 9		102.2 ± 6.76, n = 10		148.4 ± 8.10, n = 10	
	DES$_{direct-5}$ control	SNP	6.50 ± 0.07, n = 8	0.60 (paired, two-tailed t-test)	72.88 ± 7.23, n = 10	0.04 (Wilcoxon matched pairs test)	99.61 ± 10.52, n = 10	0.11 (Wilcoxon matched pairs test)
	DES$_{direct-5}$		6.45 ± 0.11, n = 8		93.15 ± 5.36, n = 10		124.5 ± 6.42, n = 10	
	Gen$_{direct-20}$ control	ACh	7.64 ± 0.07, n =7	0.002 (paired,	106.6 ± 8.36, n =7	0.05 (paired,	215.9 ± 22.44, n = 7	0.22 (paired, two-tailed t-test)

	$\text{Gen}_{\text{direct-20}}$		7.34 ± 0.09, n = 7	two-tailed t-test)	94.29 ± 7.37, n = 7	two-tailed t-test)	192.5 ± 14.44, n = 7	
	$\text{Gen}_{\text{direct-20}}$ control	SNP	6.56 ± 0.16, n = 7	0.69 (Wilcoxon matched-pairs test)	94.73 ± 5.87, n = 7	0.40 (paired, two-tailed t-test)	143.9 ± 17.36, n = 7	0.48 (paired, two-tailed t-test)
	$\text{Gen}_{\text{direct-20}}$		6.53 ± 0.11, n = 7		87.00 ± 5.27, n = 7		131.3 ± 11.50, n = 7	
Mesenteric arteries	$\text{DES}_{\text{water}}$ control	U46619	8.48 ± 0.08, n = 7	0.24 (unpaired, two-tailed t-test)	113.8 ± 5.26, n = 7	0.20 (unpaired, two-tailed t-test)	263.5 ± 12.09, n = 7	0.19 (Mann Whitney test)
	$\text{DES}_{\text{water}}$		8.62 ± 0.08, n = 8		121.3 ± 2.33, n = 8		295.5 ± 9.404, n = 8	
	$\text{DES}_{\text{water}}$ control	PE	5.86 ± 0.12, n = 7	0.41 (unpaired, two-tailed t-test)	96.25 ± 3.07, n = 7	0.30 (unpaired, two-tailed t-test)	93.83 ± 10.26, n = 7	0.08 (unpaired, two-tailed t-test)
	$\text{DES}_{\text{water}}$		6.02 ± 0.14, n = 8		101.9 ± 3.98, n = 8		122.1 ± 10.36, n = 8	

Supplementary Table 2: Gene expression normalized to the housekeeper *Rps29* for that which was significantly different for the housekeeper *Rpl13a*. Data expressed as mean ± standard error of the mean (SEM) provided with sample number (n). P values (P) provided for each statistical analysis with the type of statistical test in brackets. Significance at P < 0.05.

Sample	*Esr1*	P value (test)	*Nos3*	P value (test)	*BclXL*	P value (test)	*Bcl2*	P value (test)	*Parp1*	P value (test)	*Bak1*	P value (test)
DES$_{water}$ control	0.82 ± 0.06, n = 7	0.01 (unpaired, two-tailed t-test)	N/A									
DES$_{water}$	1.59 ± 0.22, n = 8											
Gen$_{direct\text{-}20}$ control	N/A		1.06 ± 0.16, n = 6	0.08 (unpaired, two-tailed t-test)	1.14 ± 0.23, n = 6	0.13 (unpaired, two-tailed t-test)	1.05 ± 0.16, n = 6	0.24 (Mann-Whitney test)	1.15 ± 0.26, n = 6	0.08 (unpaired, two-tailed t-test)	1.05 ± 0.13, n = 6	0.25 (unpaired, two-tailed t-test)
Gen$_{direct\text{-}20}$			0.67 ± 0.12, n = 6		0.70 ± 0.13, n = 6		0.68 ± 0.14, n = 6		0.60 ± 0.10, n = 6		0.82 ± 0.13, n = 6	

Supplementary Table 3: QuPath pixel thresholder for CC sections stained in Masson's Trichrome

Stain	Stain vector	Threshold value	Sigma value	Target tissue
Biebrich Scarlet Acid Fuchsin	0.197 0.85 0.488	0.6	3.0	Smooth muscle
Aniline Blue	0.915 0.396 0.081	1.1	0.0	Collagen
Hematoxylin	0.651 0.701 0.29	NA	NA	Nucleus

Supplementary Table 4: RT-PCR primers

Target	Forward (5' to 3')	Reverse (5' to 3')
Esr1	TCC TTC TAG ACC CTT CAG TGA AGC C	ACA TGT CAA AGA TCT CCA CCA TGC C
Ar	GTG GAT GAC CAG ATG GCG GT	GGT GCC TCA TCC TCA CAC ACT
Sfrp1	ATG ACC CCG CCC AAT ACC AC	ACT CGT TGT CGC ATG GAG GA
Tbxar2	GCT GCC GCC TGT GCT ACT TCA	GAT GTG GCC GTG GGT CGT G
Angpt4	GGG GCT CCA ATA GCC TCC AG	TGC ACC AGG TGT GAC CTC AA
Abi3bp	AGG CAA ATG CAA CAT GCT CTC CAG	TTG GAC GCA GGA ACT TCA AGA GGA
Igfbp3	AGT GAG TCC GAG GAG GAC C	GAC TCA GAG GAG AAG TTC TGG G
Tgfb2	TGC TGC CTT CGC CCT CTT TA	GGG ACA CAC AGC AAG GGG AA
Nos3	GAT GGC GAA GCG TGT GAA GG	CAC CAG TGC CTC GTG CTC TA
Oxtr	ACT GTG GTG CAA CTT CCC CG	AGG ACC CAG CCA CTT GAA GC
BclXL	TGG ATC TCT ACG GGA ACA ATG C	GTG GCT GAA GAG AGA GTT GTG G
Bcl2	GAA CCG GCA TCT GCA CAC CT	CCA GGT ATG CAC CCA GAG TGA
Parp1	CCA GCG CAG CTC AGA GAA GCC A	CAT GTT CGA TGG GAA AGT CCC
Bak1	ATG GAT CGC ACA GAG AGG CG	GTT TAG TCC AGC CAG GCA CG
Bax	CCA AGA AGC TGA GCG AGT GTC	TGA GGA CTC CAG CCA CAA AGA
Rpl13a	AAG CAG GTA CTT CTG GGC CG	ACA GTG CGC CAG AAA ATG CG
Rps29	TGA AGG CAA GAT GGG TCA C	GCA CAT GTT CAG CCC GTA TT
