# Supplementary material for: Estrogenic endocrine disruptor exposure directly impacts erectile function
Source: Commun Biol. 2024 Apr 2;7:403. doi: 10.1038/s42003-024-06048-1 (PMC10987563; doi:10.1038/s42003-024-06048-1)
Supplement: Supplementary file 4 — Reporting Summary [file 42003_2024_6048_MOESM4_ESM.pdf]

nature portfolio

Corresponding author(s): *Double-anonymous peer review submissions: write DAPR and your manuscript number here instead of author names.*

Last updated by author(s): *YYYY-MM-DD*

Reporting Summary

Nature Portfolio wishes to improve the reproducibility of the work that we publish. This form provides structure for consistency and transparency in reporting. For further information on Nature Portfolio policies, see our Editorial Policies and the Editorial Policy Checklist.

Statistics

For all statistical analyses, confirm that the following items are present in the figure legend, table legend, main text, or Methods section.

n/a	Confirmed	
☐	☒	The exact sample size (*n*) for each experimental group/condition, given as a discrete number and unit of measurement
☐	☒	A statement on whether measurements were taken from distinct samples or whether the same sample was measured repeatedly
☐	☒	The statistical test(s) used AND whether they are one- or two-sided<br>*Only common tests should be described solely by name; describe more complex techniques in the Methods section.*
☐	☒	A description of all covariates tested
☐	☒	A description of any assumptions or corrections, such as tests of normality and adjustment for multiple comparisons
☐	☒	A full description of the statistical parameters including central tendency (e.g. means) or other basic estimates (e.g. regression coefficient) AND variation (e.g. standard deviation) or associated estimates of uncertainty (e.g. confidence intervals)
☒	☐	For null hypothesis testing, the test statistic (e.g. *F*, *t*, *r*) with confidence intervals, effect sizes, degrees of freedom and *P* value noted<br>*Give P values as exact values whenever suitable.*
☒	☐	For Bayesian analysis, information on the choice of priors and Markov chain Monte Carlo settings
☒	☐	For hierarchical and complex designs, identification of the appropriate level for tests and full reporting of outcomes
☒	☐	Estimates of effect sizes (e.g. Cohen's *d*, Pearson's *r*), indicating how they were calculated

Our web collection on statistics for biologists contains articles on many of the points above.

Software and code

Policy information about availability of computer code

Data collection	ADI Instruments Lab Chart and Lab Chart Reader v. 8.1.14 used for wire myography data collection
Data analysis	Prism 9 v. 9.2.0 used to provide all statistical analyses for all parameters except histological analyses. Smooth muscle to collagen ration in CC was analysed with QuPath v. 0.3.2

For manuscripts utilizing custom algorithms or software that are central to the research but not yet described in published literature, software must be made available to editors and reviewers. We strongly encourage code deposition in a community repository (e.g. GitHub). See the Nature Portfolio guidelines for submitting code & software for further information.

Data

Policy information about availability of data

All manuscripts must include a data availability statement. This statement should provide the following information, where applicable:

- Accession codes, unique identifiers, or web links for publicly available datasets
- A description of any restrictions on data availability
- For clinical datasets or third party data, please ensure that the statement adheres to our policy

The authors declare that all the data that support the findings of this study are available within the paper [and its supplementary information files].

Research involving human participants, their data, or biological material

Policy information about studies with human participants or human data. See also policy information about sex, gender (identity/presentation), and sexual orientation and race, ethnicity and racism.

Reporting on sex and gender	NA
Reporting on race, ethnicity, or other socially relevant groupings	NA
Population characteristics	NA
Recruitment	NA
Ethics oversight	NA

Note that full information on the approval of the study protocol must also be provided in the manuscript.

Field-specific reporting

Please select the one below that is the best fit for your research. If you are not sure, read the appropriate sections before making your selection.

☒ Life sciences ☐ Behavioural & social sciences ☐ Ecological, evolutionary & environmental sciences

For a reference copy of the document with all sections, see nature.com/documents/nr-reporting-summary-flat.pdf

Life sciences study design

All studies must disclose on these points even when the disclosure is negative.

Sample size	Very few studies have assessed the corpus cavernosum as we have in this study. Based on the limited work of others and our previous work using the technique of wire myography, an n=5-8 is often sufficient to demonstrate relevant statistical differences.<br><br>Marshall, S. A., et al., The novel small-molecule annexin-A1 mimetic, compound 17b, elicits vasoprotective actions in streptozotocin-induced diabetic mice. Int J Mol Sci 2020, 21, E1384.<br><br>Marshall, S. A., et al., Relaxin deficiency leads to uterine artery dysfunction during pregnancy in mice. Front Physiol 2018, 9, 255.<br><br>Langston-Cox, A., et al., Sulforaphane improves vascular reactivity in mouse and human arteries after “preeclamptic-like” injury. Placenta 2020, 101, 242-50.
Data exclusions	Wire myography data was excluded if samples did not pass the establishment step of the protocol as the invasive nature of the technique can sometimes damage the tissue beyond possibility of analysis. This is a common practice for these protocols.RT-PCR data points were excluded if they did not pass RNA quality control checks.
Replication	All experiments are reproducible.
Randomization	For systemic DES treatments, one group of pregnant mice were randomly allocated to treatment while the other were allocated as controls within a short time frame of each other. For direct acute exposures, one CC sample from each mouse was randomly allocated to a treatment while the other was allocated to a control.
Blinding	Blinding was not relevant given that all data presented here is quantitative and not susceptible to bias.

Reporting for specific materials, systems and methods

We require information from authors about some types of materials, experimental systems and methods used in many studies. Here, indicate whether each material, system or method listed is relevant to your study. If you are not sure if a list item applies to your research, read the appropriate section before selecting a response.

Materials & experimental systems

n/a	Involved in the study
☒	☐ Antibodies
☒	☐ Eukaryotic cell lines
☒	☐ Palaeontology and archaeology
☐	☒ Animals and other organisms
☒	☐ Clinical data
☒	☐ Dual use research of concern
☒	☐ Plants

Methods

n/a	Involved in the study
☒	☐ ChIP-seq
☒	☐ Flow cytometry
☒	☐ MRI-based neuroimaging

Animals and other research organisms

Policy information about studies involving animals; ARRIVE guidelines recommended for reporting animal research, and Sex and Gender in Research

Laboratory animals	Mouse (C57BL/6). Age: postnatal day (P) 64-96
Wild animals	The study did not involve wild animals
Reporting on sex	Findings apply only to males as we are modeling a condition that occurs in men (erectile dysfunction).
Field-collected samples	The study did not involve samples collected from the field.
Ethics oversight	University of Melbourne Animal Ethics Committee

Note that full information on the approval of the study protocol must also be provided in the manuscript.

Plants

Seed stocks	NA
Novel plant genotypes	NA
Authentication	NA
